# Pharmacogenomic Stratification for Oncology Drug Repurposing: An Exposure Target Context Eligibility Framework

**DOI:** 10.3390/ph19060957

**Published:** 2026-06-19

**Authors:** Mohamed El-Tanani, Adil Farooq Wali, Syed Arman Rabbani, Yahia El-Tanani, Imran Rangraze, Frezah Muhana

**Affiliations:** 1 RAK College of Pharmacy, Ras Al Khaimah Medical and Health Sciences University, Ras Al Khaimah P.O. Box 11172, United Arab Emirates; farooq@rakmhsu.ac.ae (A.F.W.);; 2 Royal Cornwall Hospital Trust, National Health Service, Truro TR1 3LJ, UK; 3 RAK College of Medical Sciences, Ras Al Khaimah Medical and Health Sciences University, Ras Al Khaimah P.O. Box 11172, United Arab Emirates; 4 Princess Sarvath Community College, Amman 11196, Jordan

**Keywords:** drug repurposing, pharmacogenomics, precision oncology, predictive biomarkers, ADME, tumor microenvironment, R = E × T × C, PSDR

## Abstract

In spite of plausible biology, the majority of oncology trials involving drug repurposing have failed to demonstrate any efficacy. Numerous factors can potentially cause failure, including issues with dosing, drug strength, the trial design itself, and patient diversity. A major, potentially correctable contributor is the absence of pharmacogenomic eligibility criteria. Here, we propose the Pharmacogenomic Stratification Framework for Drug Repurposing (PSDR), a novel framework for drug response that encompasses the triad of Exposure (E, pharmacokinetic adequacy), Target engagement (T, somatic tumor genomics), and Context competence (C, tumor microenvironment). These domains are represented as R = E × T × C, an eligibility model capturing the necessary, though not sufficient, conditions for anticancer drug activity. The model is not presented as an empirically validated quantitative law but as a conceptual framework to guide biomarker-stratified trial design. We derive five testable pharmacogenomic hypotheses for metformin, statins, beta-blockers, NSAIDs, and SSRIs, and propose a three-point PSDR eligibility scoring system. Prospective validation of each hypothesis in appropriately stratified cohorts is required before clinical implementation. The PSDR framework complements rather than replaces existing precision oncology resources (OncoKB, CIViC, PharmGKB, CPIC, DepMap, GDSC) by integrating germline pharmacokinetics, somatic genomics, and microenvironmental profiling for repurposed agents. If validated, PSDR-guided enrichment designs could substantially improve the efficiency and interpretability of repurposing trials. The PSDR framework should be considered a conceptual and hypothesis-generating model that requires prospective validation before clinical implementation.

## 1. Introduction

Drug repurposing—the identification of new oncological indications for approved or investigational compounds—occupies a distinctive position in cancer drug development. Repurposed agents enter trials with established safety profiles, known pharmacokinetic properties, and often decades of clinical exposure data. The cost and timeline advantages over de novo drug discovery are substantial [1,2,3]. Metformin, statins, beta-blockers, NSAIDs, and selective serotonin reuptake inhibitors have each generated epidemiological signals of anticancer activity, and their repurposing has been pursued across hundreds of clinical trials [4,5,6].

The clinical record is, by any measure, disappointing. Large, well-powered randomized trials, including the MA.32 trial of metformin in breast cancer, multiple statin trials across tumor types, and observational to interventional translations of beta-blocker and NSAID data, have failed to demonstrate meaningful survival benefits in unselected populations [7,8]. This pattern has been attributed to various causes: inadequate dosing, off-target pharmacology, tumor heterogeneity, trial design limitations, and, in some cases, genuine absence of anticancer activity. These explanations are not wrong. But they are incomplete.

One consistent and correctable structural limitation is how these trials are designed. Most of these “repurposing trials” are conducted without any stratification based on pharmacogenomics. Therefore, patients are entered into trials on the basis of their tumor type and performance status (indicative of a patient’s potential to withstand chemotherapy) rather than on the basis of a genotype that would predict benefit from a particular medication. Many of these drugs have genotype-specific effects on cancer growth, and thus, conducting trials without such stratification is more than a minor limitation of how these studies are set up; it is an approach that may substantially dilute the ability to detect clinically meaningful treatment effects in biologically responsive subgroups.

An additional factor to consider is how most targeted cancer therapies are designed and tested within the framework of specific biomarkers. EGFR inhibitors, for example, were first found to have activity in non-small cell lung cancer patients who were EGFR mutation-positive [9] and subsequently similar therapies were developed for additional cancer types. In some cases, preclinical data first demonstrated efficacy of a compound targeting a particular biomarker, followed by clinical testing in patients deficient in that biomarker. While such agents have not proven to be dramatically more effective than the large library of existing repurposable therapies, they have at least been tested in the right patient population [10].

This review outlines the Pharmacogenomic Stratification Framework for Drug Repurposing (PSDR), which describes drug response as a multiplicative function of exposure, target engagement and pathway context, which is generalizable to all drug classes, tumor types and therapeutic strategies. The framework for drug repurposing trial design, termed Pharmacogenomic Stratification for Drug Repurposing (PSDR), is operationalized as a three-pillar decision logic (3D) referred to as PSDR 3D. PSDR 3D integrates germline variation influencing drug ADME with somatic tumor genomics as well as tumor microenvironment (TME) heterogeneity to define molecular trial eligibility. While pharmacogenomics is not the sole reason for failures in drug repurposing, pharmacogenomic stratification is a necessary condition for the conduct of a trial and for generating meaningful results from said trial.

The objective of this review is not to establish PSDR as a validated predictive model but rather to present a conceptual framework that integrates existing pharmacokinetic, genomic, and microenvironmental knowledge into a unified eligibility structure. The framework is intended to generate testable hypotheses and guide future biomarker-enriched clinical trial designs. Its clinical utility and predictive performance remain to be established through prospective validation studies.

### Literature Selection Approach

This manuscript was prepared as a narrative conceptual review rather than as a systematic review. Relevant literature was identified through targeted searches of PubMed, Scopus, Web of Science, Google Scholar, and major pharmacogenomic resources including PharmGKB, CPIC, OncoKB, CIViC, DepMap, and GDSC. Studies were selected to illustrate key concepts relevant to pharmacogenomics, drug repurposing, biomarker-guided trial design, and tumor microenvironment biology. The examples presented were chosen for their mechanistic relevance and illustrative value and should not be interpreted as a comprehensive or systematic representation of all available evidence.

## 2. The PSDR Framework: A Conceptual Shift in Oncology Drug Development

### 2.1. What the Field Was Missing Before PSDR

The central claim: Repurposed drugs are not non-specific agents. They have genotype-dependent mechanisms. Testing them without molecular eligibility criteria is not a neutral design choice and is a likely contributor to null or attenuated treatment effects [11].

A common unconscious assumption in current drug repurposing efforts in oncology is that the anticancer activity of a known drug will be relevant to most, if not all, patients with a given type of cancer. The traditional drug repurposing paradigm was based on the now-outmoded model of cytotoxic chemotherapy where a “cytotoxic” agent (i.e., a drug that is intentionally toxic) kills rapidly dividing cells, often through a mechanism that is independent of the patient’s germline genome sequence and the specifics of their tumor. However, many known drugs have alternative mechanisms of action that depend on the function of a particular drug transporter, the availability or activity of a specific biological pathway, or the characteristics of a particular tumor microenvironment.

Metformin requires active uptake by OCT1/2 transporters to accumulate intracellularly; its primary anticancer mechanism, AMPK activation via LKB1, is non-functional in LKB1-mutant tumors, which constitute 15–30% of lung adenocarcinomas [12]. Importantly, the proposed LKB1-dependent framework does not exclude the possibility of alternative AMPK-independent mechanisms. Metformin may exert anticancer effects through modulation of mitochondrial respiration, systemic insulin signaling, or other metabolic pathways that remain incompletely characterized. Consequently, LKB1 status should be regarded as a probabilistic rather than absolute determinant of response. Statins require SLCO1B1-mediated hepatic influx for systemic exposure; their proapoptotic effects depend on intact TP53 signaling, which is lost in approximately 50% of human cancers [13]. Future studies should stratify TP53 alterations according to functional class (loss-of-function, dominant-negative, or gain-of-function mutations) rather than treating TP53 status as a binary variable. Beta-blockers modulate sympathetic signaling in the tumor microenvironment; their efficacy requires both ADRB2 receptor sensitivity and a sympathetically innervated TME [14]. NSAIDs inhibit COX-2 in a pathway that is selectively dependency-forming in PIK3CA-mutant tumors [15].

None of these dependencies are obscure. All are mechanistically well-characterized. Yet none have been used as enrollment criteria in the trials that tested these drugs. The field has treated agents with genotype-dependent mechanisms as though they were non-specific, and then attributed the resulting null results to biological implausibility.

What was missing before PSDR was not data; it was a structured framework for translating available pharmacogenomic knowledge into explicit eligibility criteria, and a unifying principle that explains why such criteria are not merely useful but logically necessary.

### 2.2. PSDR as a Conceptual Shift, Not an Incremental Refinement

The standard approach to drug development focuses on determining whether a small molecule has anticancer activity. In contrast, the Pharmacogenomic Stratification Framework for Drug Repurposing (PSDR) approach seeks to find the question prior to this one: for which patients (defined by germline genotype, somatic tumor alterations, and/or microenvironment) does a drug have a mechanistic basis for activity. The PSDR approach thereby transforms what had been an administrative criterion for inclusion in a clinical trial—patient eligibility—to a scientific question that can be addressed.

The multiplicative formulation should not be interpreted as a validated quantitative equation. Rather, it represents a conceptual framework intended to capture the conditional and sequential nature of drug activity. Whether multiplicative models outperform additive, logistic, or alternative predictive frameworks remains an empirical question requiring prospective investigation.

The failure of a repurposing trial in an unselected group of patients with a particular type of cancer is frequently taken as evidence that the drug in question does not have anticancer activity. This is not evidence of no anticancer activity; it is evidence of no anticancer activity in an undefined, somewhat heterogeneous, unselected group of patients, and therefore has little, if any, actual scientific content.

Moreover, since positive epidemiological signals are most likely to derive from the subset of patients for whom pharmacogenomics can define optimal levels of therapy, failure to account for this signal enrichment in subsequent interventional studies will inevitably lead to negative results.

Third, there is already existing and paid for critical infrastructure in place to conduct pharmacogenomically stratified repurposing trials. The CPIC guidelines and PharmGKB bioinformatics databases have been developed and paid for to guide the use of genetic information to inform drug selection, dosing, and monitoring. In addition, many commercial platforms are now incorporating germline genotyping into standard clinical testing, and several tumor genomic profiling platforms are beginning to be used to select patients and stratify trials. The only remaining barrier to leveraging this pre-existing investment is to make a conceptual leap to incorporate these tools.

### 2.3. An Exposure–Target–Context Eligibility Model for Drug Response

#### 2.3.1. The Eligibility Principle

A widespread misconception in drug development is that small molecule drug efficacy is a property of the drug molecule itself. Drug efficacy is better understood as conditional on pharmacogenomic context. In reality, efficacy is a property of the drug-molecule–patient interaction. This crucial reality derives from three key variables, each of which are (1) independent, (2) non-substitutable and (3) crucial. Namely, (1) to what extent does the drug actually reach the site of action? (2) To what extent do cells of the patient have present and functional the target molecule to which the drug is meant to bind? (3) To what extent is the downstream pathway that follows from binding of the target molecule to the drug active and capable of delivering an appropriate therapeutic effect? While a potent mechanism of action, impressive preclinical activity and a strong pharmacogenomic rationale can all matter, not one single small molecule drug has reached market in the absence of all of these variables. This is a property of biology that trumps all else.

This principle is not new in its components. Pharmacokinetics has long established that exposure determines concentration at the target site. Pharmacogenomics has demonstrated that germline and somatic variation determines target availability and pathway function. What is new is the explicit formulation of these variables as multiplicative, not additive, determinants of response, and the recognition that this multiplicative structure has direct, unavoidable consequences for trial design.

PSDR operationalizes this principle for the specific challenge of drug repurposing in oncology, where all three variables are poorly characterized for agents that were never developed with oncology biomarkers in mind [16,17,18]. But the principle itself is general. It applies to targeted therapies, immunotherapies, and conventional cytotoxic agents with equal logical force.

#### 2.3.2. The Formal Model: R = E × T × C

Drug response can be expressed as a multiplicative function of three distinct but interacting domains:

R = E × T × C

where:

- R = Drug response (probability of clinically meaningful anticancer effect);
- E = Exposure (pharmacokinetic adequacy, or the degree to which therapeutic drug concentrations are achieved at the tumor site, determined by germline ADME variation: uptake transporters, metabolic enzymes, efflux pumps);
- T = Target engagement (the degree to which the drug’s molecular mechanism has a biologically functional target in the tumor, determined by somatic genomic profile: pathway-defining mutations, copy number alterations, fusion genes);
- C = Context competence (the degree to which the tumor microenvironmental context supports downstream pathway transduction of the drug’s effect, determined by TME composition: immune infiltration, metabolic phenotype, stromal architecture, sympathetic innervation).

Each variable is conceptualized on a continuous scale from 0 (complete absence of the condition) to 1 (full presence). The multiplicative structure means that all three domains must be non-zero for meaningful response. It is important to note that E, T, and C are distinct but interacting domains: tumor genotype can shape microenvironmental features, and inflammatory context can regulate transporter expression. This biological correlation is acknowledged as a limitation of treating the domains as fully separable.

**Figure 1:**
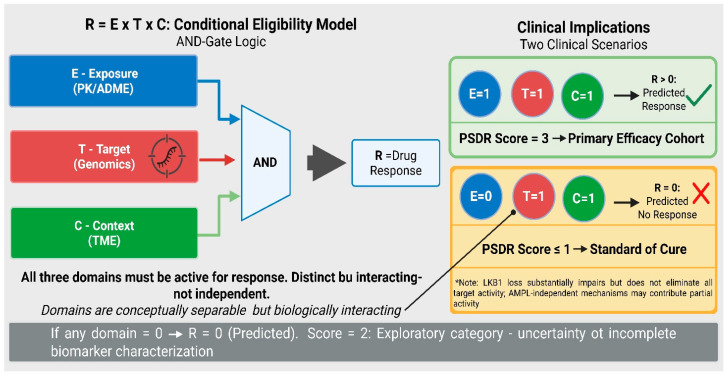
Drug response is conditional: R = E × T × C. Three distinct but interacting domains—E (Exposure, blue), T (Target, red), and C (Context, green)—converge on an AND gate (left). Response requires all three inputs simultaneously. Right: two cases. Case 1: E = 1, T = 1, C = 1 → R > 0 (response). Case 2: E = 0, T = 1, C = 1 → R = 0 (no response). Bottom rule: if any condition = 0 → No response.

- If E = 0 (drug does not reach the tumor): R = 0, regardless of T and C;
- If T = 0 (no functional molecular target): R = 0, regardless of E and C;
- If C = 0 (pathway context is incompetent): R = 0, regardless of E and T;
- Only when E > 0, T > 0, and C > 0 is meaningful response possible. This conditional relationship is illustrated in Figure 1.

This formulation captures a key conditional dependency that additive models may not fully capture: pharmacogenomic variables do not simply compensate for each other in the way an additive structure would imply. A patient with perfect drug exposure but a non-functional molecular target does not achieve partial response—they achieve no mechanistic response. The multiplicative structure is not a modeling convenience; it reflects the sequential, conditional logic of drug action at the molecular level.

#### 2.3.3. The PSDR Eligibility Scoring System

For clinical application, the continuous R = E × T × C model is operationalized as a discrete three-point eligibility scoring system:

PSDR Score = E_score + T_score + C_score

Thresholds defining E, T, and C eligibility are expected to vary according to drug class, tumor type, biomarker assay, and clinical setting. Prospective calibration studies will therefore be required before standardized implementation can be considered. The PSDR scoring system and its clinical interpretation are outlined in Table 1.

Where each component is scored:

- 1 = Eligible (biomarker assessment predicts adequate exposure/functional target/competent context);
- 0 = Ineligible (biomarker assessment predicts inadequate exposure/absent target/incompetent context).

Score Interpretation:

PSDR Score = 3: All three eligibility domains met → primary efficacy cohort (enroll in biomarker-enriched trial arm; powered for primary endpoint).

PSDR Score = 2: Two domains met, one scored 0 or uncertain → exploratory/mechanistic cohort only (not powered for primary efficacy; hypothesis-generating secondary analyses only).

PSDR Score ≤ 1: One or zero domains met → predicted low eligibility (standard-of-care or alternative trial).

Note: Binary 0/1 scoring is a practical approximation under clinical uncertainty. True eligibility exists on a continuum; thresholds require prospective calibration for each drug–biomarker combination. Score = 2 reflects uncertainty or incomplete biomarker characterization rather than true absence of a domain; it is therefore treated as an exploratory eligibility category.

The binary scoring framework is intentionally simplified to facilitate conceptual illustration and trial design discussions. In biological systems, exposure, target engagement, and microenvironmental competence exist along continuous rather than dichotomous scales. Consequently, the proposed score should not be interpreted as a deterministic predictor of response. Future implementations may require probabilistic weighting, continuous biomarker integration, or machine-learning approaches capable of capturing nonlinear interactions among eligibility domains.

Trial design application: The proposed cohort structure is presented as an illustrative example of how PSDR could be operationalized in future biomarker-enriched studies. These recommendations should not be interpreted as validated clinical eligibility criteria. Prospective studies will be required to determine whether PSDR-based enrichment improves predictive performance, statistical efficiency, or clinical outcomes compared with conventional trial designs. A potential consequence of successful biomarker enrichment is improved statistical efficiency relative to unselected populations. However, the magnitude of any reduction in sample-size requirements remains unknown and would depend on biomarker prevalence, effect size, assay performance, and trial design characteristics. Formal modeling studies will be required before quantitative claims regarding sample-size reduction can be made.

#### 2.3.4. The Eligibility Statement

“Drug response is conditional on three necessary domains: exposure, target engagement, and pathway context. When any domain is absent or severely impaired, clinically meaningful response is unlikely regardless of the status of the other domains. This is the eligibility principle underlying the PSDR framework.”

This statement is a testable eligibility framework grounded in established pharmacological principles. It formalizes relationships that have been implicit in pharmacology for decades. PSDR makes it explicit, operationalizes it, and applies it to a drug class where it has been systematically ignored.

It is important to distinguish between the logical prerequisites, measurable biomarkers, causal variables, and predictive models that together constitute the PSDR framework. E, T, and C are defined as necessary conditions for drug response: their absence makes response unlikely by definition. This is a logical (eligibility) statement, not an empirical discovery. The framework becomes empirically valuable when E, T, and C are operationalized as measurable biomarkers—OCT1/2 genotype, LKB1 status, metabolic phenotype—that can be prospectively tested as predictors of response in stratified clinical trials. The distinction between logical prerequisites and predictive biomarkers is acknowledged as a limitation of the current framework.

A corollary follows directly: “An unselected repurposing trial is not a test of whether a drug works. It is a test of whether a drug works in a population defined by unknown and unmeasured eligibility criteria a question with a population-average estimate under tested conditions.”

#### 2.3.5. Generalization Beyond Repurposed Drugs

The R = E × T × C eligibility model is not unique to repurposed drugs. It describes a general conceptual framework for understanding the conditions necessary for drug activity.

In contrast to the “magic bullet” notion of targeted therapy, EGFR inhibitors require three key elements for efficacy: E—enough drug exposure (concentration); T—a tumor with an activating EGFR mutation; and C—no alternative bypass mechanisms. All trials in this class have shown success because all have systematically evaluated these three criteria, and nothing to do with the superior quality of the drugs involved.

Immunotherapy involves (E)xposure to drug, (T)argeting of drug to tumor or immune cells, and manipulation of the cancer tumor immune microenvironment that is not irreversibly excluded or suppressed. In current studies, TMB, PD-L1 expression, and tumor-infiltrating lymphocyte density serve as imperfect but real surrogates for T and C. However, there is significant variation in outcomes to immunotherapy because we do not have a complete understanding of E, T, and C.

In addition to the variables that influence mechanism-dependent agents, even the so-called mechanism-independent agents such as the conventional cytotoxics (the alkylating agents and the antimetabolites) show pharmacogenomic variability. Variants in the DPYD gene predict for susceptibility to severe toxicity from fluorouracil (E). The methylation status of the MGMT gene predicts for target competence of temozolomide in glioblastoma (T). In addition to effects on the drug itself, tumor hypoxia and its vascular architecture (C) determine drug penetration. The variables are less well characterized for the conventional agents but the same rules apply.

PSDR contrasts the commonly held belief that there are specific tools and techniques that can be used for particular tasks in drug response biology with the finding that there is a general principle that underlies many of the currently used approaches—including those that incorporate pharmacokinetic, pharmacogenomic and tumor biology information—which have never been implemented in a unified fashion that is easy to use and that interact in a multiplicative fashion.

#### 2.3.6. Why Drug Response Must Be Multiplicative

The multiplicative interaction term R = E × T × C reflects the biological logic that all three necessary conditions must be met. It is a conceptual choice that follows from representing in the model the sequential and conditional relationships between variables that the model includes. That is, looking at each of the variables in isolation, if E = 0, then there is no drug effect because the drug does not reach its target. If T = 0, then there are no effects related to the amount of drug (E) because pharmacology does not come into the matter regardless of dose. And if C = 0, then the effect of E × T has no effects downstream of the binding of drug to target. Every time someone says “except for X,” it is almost always because X was not factored into the equation in the first place. There is usually an unconsidered variable preventing a proper explanation.

Additive models tend to oversimplify the conditional relationship between exposure, target engagement, and context with respect to drug action. Logically, in a strictly additive model, exposure to the drug at high levels could offset low target engagement and still achieve downstream response, something that is highly unlikely when target engagement is a prerequisite for action. Conversely, exposure, target engagement and pathway competence in the R = E × T × C model are treated as sequential conditions to a drug’s action and, therefore, represent the logical conditions to a drug threshold. The effect of the drug in this context is only achieved and sustained when all three elements are present and sufficient. Whether this type of logic—more specifically, the multiplicative model—is preferable to the additive and/or logistic models in predictive capability is a question for scientific research that mandates structured models of trial data delineated by tiers.

### 2.4. Pillar 1—Pharmacokinetic Adequacy: Germline ADME Variation

Best-in-class molecules mean nothing if the quantity delivered to the relevant tissues and organs is suboptimal. In designing a small molecule drug, achieving adequate PK is typically the primary objective. Why develop a great small molecule if it does not reach the active site to engage with its target? If E = 0 (drug exposure at the active site is negligible), then R = 0 regardless of T and C. PK exposure to the active site is an essential driver of small molecule drug performance. Many drugs do not act because they do not reach their site of action because of the way the body ‘disposes’ of them. The body’s ‘disposition’ of a drug is significantly affected by the genes that are inherited from our parents. Three groups of genes can affect the disposition of a drug: those that influence its absorption into the bloodstream; those that encode the enzymes responsible for its biotransformation to more water-soluble metabolites; and those that influence its excretion from the body. The aggregate effect of these genes determines the efficacy and exposure of a drug. Some genes can affect how we take up a medicine in our gut. For example, the gene OCT1 has versions that affect how people take up the common diabetes medicine metformin—some people take it up poorly, whereas others take it up efficiently. Similarly, genes process some medicines slowly in the liver, causing their levels to build up to potentially toxic amounts. For example, different versions of the CYP2D6 gene process certain medicines slowly. Another gene that affects uptake of a medicine in the liver is SLCO1B1. This gene has versions that affect the uptake of cholesterol-lowering medicines, which is why some people do not get adequate benefit from these medicines. By recognizing these differences we can find more effective treatments for individual patients. Representative examples of E, T, and C eligibility criteria for major repurposed drug classes are summarized in Table 2.

The clinical implication is direct: germline PK genotyping must precede enrollment in repurposing trials as a primary eligibility criterion—not as a pharmacovigilance measure. Patients with E = 0 (predicted subtherapeutic tumor exposure) are not candidates for efficacy assessment; their inclusion dilutes the trial signal and produces uninterpretable results.

### 2.5. Pillar 2—Target Engagement: Somatic Tumor Genomics

Drug exposure to tumor cells is a necessary but not sufficient component for anticancer activity; the drug must also interact with a relevant target within the cancer cell. When evaluating a repurposed agent originally developed for a non-oncologic indication, the relevant oncologic molecular target is typically a metabolic enzyme, signaling kinase, or pathway node that may be either mutated or otherwise aberrantly expressed [19,20,21,22,23].

Mutations in oncogenes and tumor suppressor genes can result in a tumor with specific dependencies on different bioactive pathways. Mutations in the tumor suppressor gene LKB1/STK11 determine dependency on metformin’s effect on the AMPK pathway. TP53 mutation determines dependency on statin-induced apoptosis, specifically through pathways involving BAX/PUMA/NOXA. PIK3CA wild-type status (and in many cases amplification) determines dependency on NSAID-mediated disruption of the PI3K pathway via prostaglandin, a pathway that NSAIDs exploit for their anti-inflammatory activity.

### 2.6. Pillar 3—Pathway Competence: Tumor Microenvironment Heterogeneity

The third variable, C, refers to the contribution of the microenvironmental tumor cellular context to the effect of the drug on the patient. Like the other two variables, this contribution is not predicted by PK or genomic profiling. Hence, even if E > 0 and T > 0, C = 0 can be the determining factor making R = 0, even when the three other variables are positively affecting tumor response to the drug. The specific context of the microenvironment and the tumor cells that are present can influence the response to drugs that are sensitive to the context in which they act.

For beta-blockers, C primarily depends on the density of sympathetic innervation of the tumor. Many tumors do not contain sympathetic nerve fibers marked by TH, rendering them insensitive to beta-blockers. The C is conceptually separable from, though potentially interacting with, tumor genotype and the particular drug used [24,25,26,27].

For metformin, C primarily depends on the metabolic properties of the tumor. Many tumors exhibit increased glycolysis driven by HIF-1α to upregulate the Warburg effect, meaning that they have little to no capacity for inhibiting OXPHOS. As a result, C ≈ 0 for metformin, which affects complex I through several distinct mechanisms.

For NSAIDs, C primarily depends on both the amount of stromal COX-2 expression and the properties of prostaglandin signaling within the TME. In an environment in which there is no COX-2, there is no prostaglandin substrate for inhibition and C ≈ 0.

TME profiling by IHC, transcriptomic deconvolution or single cell sequencing thus constitutes a third distinct domain that cannot be predicted from germline or somatic data. This third variable, designated ‘C’ here, is overlooked in the PSDR equation and has not been considered in repurposing trials to date.

### 2.7. The PSDR Decision Pathway: A Three-Step Eligibility Logic

**Figure 2:**
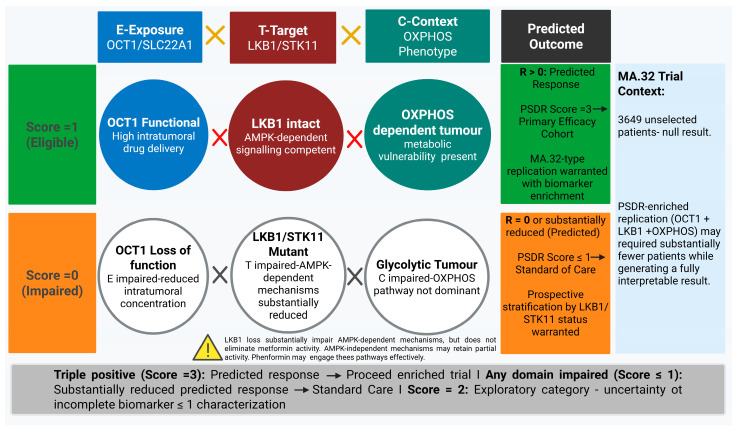
PSDR: three-step eligibility framework. A vertical decision flowchart operationalizes R = E × T × C as a sequential three-gate filter. Patients enter at “Patient enrolled” and pass through three decision diamonds: E (Exposure/PK), T (Target/Genomics), and C (Context/TME). A ✔ eligible path continues downward at each gate; an ✗ failure exits right to a single shared non-responder box. Only patients clearing all three gates (E = 1 × T = 1 × C = 1) reach the ✔ responder outcome. Bottom rule: if any step fails → non-responder.

The PSDR Decision Pathway follows a three-step eligibility logic to determine whether PSDR applies. The sequential PSDR eligibility workflow is shown in Figure 2.

PSDR Clinical Practices can further elucidate the variables in the equation R = E × T × C by operationalizing each of the variables in a three-step, sequential eligibility filter, starting with the variable most commonly a cause of ineligibility and progressing to less common causes of ineligibility as you move through the process. Germline ADME genotyping (E) is the least expensive and most scalable of the three variables and should be performed first in the PSDR Decision Pathway. Tumor somatic profiling (T) is moderately more expensive and is the second variable to be evaluated. TME (C) is the most expensive of the three variables.

The PSDR Three-Step Decision Pathway:

Step 1—Pharmacokinetic Adequacy (Score E): Genotype ADME genes relevant to the candidate drug (CYP450 enzymes, SLC uptake transporters, ABC efflux pumps). Predict drug exposure at the tumor site from genotype. E = 1 (ELIGIBLE): wild-type or gain-of-function ADME genotype → proceed to Step 2. E = 0 (INELIGIBLE): LOF variant predicting subtherapeutic tumor exposure → PSDR Score ≤ 1; exclude from efficacy cohort.

Step 2—Target Engagement (Score T): Profile tumor for pathway-defining somatic mutations (LKB1/STK11, TP53, PIK3CA, KRAS, CRBN, ADRB2 expression). Confirm drug mechanism has a functional molecular target. T = 1 (ELIGIBLE): intact pathway dependency → proceed to Step 3. T = 0 (INELIGIBLE): loss of pathway target → PSDR Score ≤ 1; exclude from efficacy cohort.

Step 3—Pathway Competence (Score C): Assess TME features relevant to drug mechanism (sympathetic innervation, immune infiltration, metabolic phenotype, stromal composition, COX-2 expression). Confirm downstream pathway is functionally active in the microenvironmental context. C = 1 (ELIGIBLE): TME supports drug mechanism engagement → PSDR Score = 3; enroll in primary efficacy cohort. C = 0 (INELIGIBLE): TME incompetent for drug mechanism → PSDR Score ≤ 1; exclude from efficacy cohort.

### 2.8. Testable Pharmacogenomic Hypotheses Derived from PSDR

The R = E × T × C formulation generates specific, falsifiable hypotheses by identifying the biomarker conditions under which each variable equals 1. Each hypothesis defines a PSDR Score = 3 patient subpopulation, a predicted clinical endpoint, and a trial design capable of testing the prediction.

**Hypothesis** **1.** *Metformin Anticancer Activity Is Restricted to OCT1-Proficient, LKB1-Intact, OXPHOS-Dependent Tumors (PSDR Score = 3)*.

R = E × T × C formulation:

- E = 1: OCT1/2 wild-type → therapeutic intracellular metformin concentration achieved;
- T = 1: LKB1/STK11 wild-type → AMPK activation competent;
- C = 1: OXPHOS-dependent tumor metabolic phenotype → complex I inhibition cytotoxic.

Predicted response: High intratumoral metformin activates AMPK, induces metabolic stress, suppresses mTORC1, and inhibits tumor growth.

Predicted null or reduced response: OCT1 loss-of-function (E impaired) or LKB1/STK11 mutation (T impaired) or glycolytic tumor phenotype (C impaired) → R is predicted to be substantially reduced. However, AMPK-independent metformin mechanisms (e.g., inhibition of mitochondrial complex I via AMPK-independent pathways, reduction in circulating insulin/IGF-1) may contribute partial activity even in LKB1-deficient tumors. Phenformin, a more potent biguanide, may engage AMPK-independent mechanisms more effectively.

**Figure 3:**
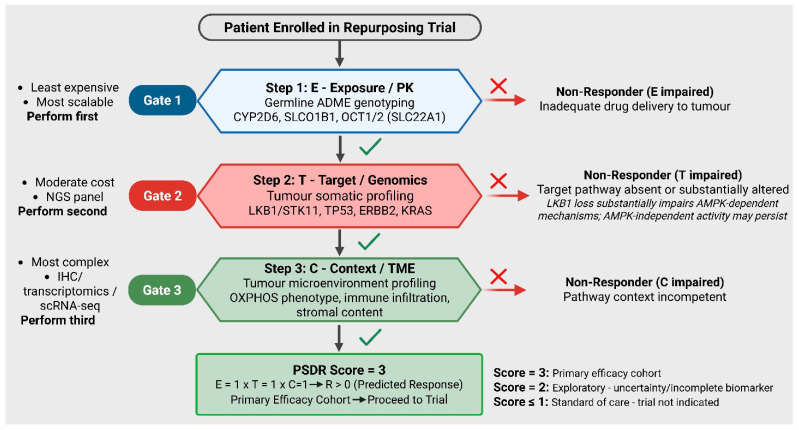
Applying R = E × T × C: metformin. Three columns represent E (Exposure, blue), T (Target, red), and C (Context, green). Each column shows a large colored circle (Score = 1, eligible) above a gray circle (Score = 0, ineligible), separated by × operators. Column labels: OCT1 (E), LKB1 (T), OXPHOS (C). Top row (Score = 1 across all three) → R > 0 (response). Bottom row (Score = 0 in any column) → R = 0 (no response). Bottom rule: triple positive → response|any negative → no response.

The metformin-specific application of the PSDR model is illustrated in Figure 3**,** which maps OCT1/2-mediated exposure, LKB1-dependent target competence, and OXPHOS-dependent context onto the E × T × C framework.

Trial design implication: The MA.32 trial enrolled 3649 unselected patients. A PSDR Score = 3 enriched replication—screening for OCT1 genotype, LKB1 status, and metabolic phenotype—may require substantially fewer patients while generating a fully interpretable result. Failure in this selected population would constitute genuine evidence of biological implausibility. Success would rescue a clinically useful agent and validate the PSDR scoring system.

**Hypothesis** **2.** *Statin Anticancer Activity Requires SLCO1B1-Mediated Exposure and TP53-Competent Apoptosis (PSDR Score = 3)*.

R = E × T × C formulation:

- E = 1: SLCO1B1 wild-type → adequate hepatic and intratumoral statin exposure;
- T = 1: TP53 wild-type → p53-dependent apoptosis competent;
- C = 1: Mevalonate-dependent tumor (high isoprenoid pathway activity).

Predicted response: Mevalonate pathway inhibition suppresses Ras prenylation and activates p53-dependent apoptosis.

Predicted null or reduced response: SLCO1B1*5 (E impaired → reduced intratumoral statin exposure) or TP53-mutant tumor (T impaired → p53-dependent apoptosis pathway altered). However, TP53 effects on statin sensitivity are allele-specific and context-dependent: gain-of-function TP53 mutations may upregulate mevalonate pathway flux, potentially sensitizing tumors to statin treatment through a distinct mechanism. Binary exclusion based on TP53 mutation status is therefore not warranted; prospective stratification by TP53 allele type is recommended.

Trial design implication: In an unselected cancer population, TP53 is mutated in ~50% and SLCO1B1\*5 is present in ~15–20%. The PSDR Score = 3 fraction is approximately 40–42%. Statin trials in unselected populations test a drug in a majority non-responder population by design. A stratified trial in SLCO1B1-WT, TP53-WT patients—embedded within NCI-MATCH or TAPUR platforms—would provide the first properly powered test of the statin repurposing hypothesis.

**Hypothesis** **3.** *Beta-Blocker Anticancer Efficacy Requires ADRB2 Sensitivity and Sympathetic TME Enrichment (PSDR Score = 3)*.

R = E × T × C formulation:

- E = 1: ADRB2 Arg16 genotype → high β2-adrenergic receptor sensitivity;
- T = 1: β2-AR expressed on tumor and immune cells → receptor target present;
- C = 1: High TH+ sympathetic nerve density in TME → adrenergic signaling substrate present.

Predicted response: β-adrenergic blockade in sympathetically innervated TME reduces VEGF, decreases MDSC infiltration, and restores CD8+ T cell activity.

Null response: ADRB2 Gly16 (E reduced) or sympathetically depleted TME (C = 0) → R = 0 or R ≈ 0.

Trial design implication: Retrospective analyses of beta-blocker use in ovarian and breast cancer show heterogeneous effect sizes consistent with TME-dependent response [6,28]. A prospective trial stratifying by TH+ sympathetic nerve density (IHC-based scoring) and ADRB2 genotype would be the first properly powered test of the sympathetic oncology hypothesis.

**Hypothesis** **4.** *NSAID/Aspirin Efficacy Is Selective for PIK3CA-Mutant, COX-2-Overexpressing Tumors (PSDR Score = 3)*.

R = E × T × C formulation:

- E = 1: CYP2C9 wild-type → normal celecoxib/aspirin metabolism; adequate exposure;
- T = 1: PIK3CA activating mutation → prostaglandin-PI3K survival dependency present;
- C = 1: COX-2 overexpression in tumor and stroma → prostaglandin signaling substrate present.

Predicted response: COX-2 inhibition depletes PGE2, disrupting PI3K-dependent survival signaling selectively in PIK3CA-mutant tumors.

Null response: PIK3CA-WT (T = 0) or COX-2-negative TME (C = 0) → R = 0.

Trial design implication: The PIK3CA-aspirin interaction (HR 0.54 in PIK3CA-mutant vs. HR 1.06 in PIK3CA-WT colorectal cancer) is among the strongest pharmacogenomic predictors identified for any repurposed drug [29]. A prospective PIK3CA-stratified analysis within the Add-Aspirin trial (NCT02804815) would provide definitive validation and establish PIK3CA mutation as the first companion diagnostic for a repurposed oncology agent.

**Hypothesis** **5.** *(Exploratory Future Hypothesis:) SSRI Repurposing in B-Cell-A Hypothesis Requiring Primary Mechanistic Evidence (PSDR Score = 3 if Validated)*.

*This example is included solely as a hypothesis-generating illustration of how the PSDR framework may be applied to future drug-repurposing opportunities. Unlike the preceding examples, direct mechanistic evidence supporting SSRI-mediated CRBN–IKZF signaling remains limited, and the proposed pathway should be regarded as exploratory until validated experimentally*.

R = E × T × C formulation:

- E = 1: CYP2D6 IM/EM → adequate SSRI plasma and intratumoral exposure;
- T = 1 (speculative): CRBN wild-type + IKZF1/3-dependent tumor → putative degradation target. Note: CRBN-mediated IKZF1/3 degradation is an established mechanism for immunomodulatory drugs (IMiDs/CELMoDs, e.g., lenalidomide, iberdomide). Direct evidence for SSRI-mediated CRBN–IKZF engagement is preliminary and requires primary mechanistic validation before clinical application;
- C = 1: B-cell lymphoma microenvironment → IKZF dependency context present.

Speculative predicted response: If SSRIs engage CRBN-mediated IKZF1/3 degradation (a mechanism currently established only for IMiDs/CELMoDs), this could induce apoptosis in IKZF-dependent B-cell malignancies. This mechanism requires direct experimental validation for SSRIs before it can be cited as an established anticancer pathway.

Predicted null or reduced response (speculative): CYP2D6 poor metabolizer (E → altered exposure/toxicity) or CRBN-mutant tumor (T impaired) or non-lymphoid tumor (C impaired) → R predicted low. These predictions are hypothesis-generating and require primary experimental validation.

Trial design implication: This hypothesis restricts SSRI repurposing to a biologically coherent but narrow indication. Testing in unselected solid tumors—as has occurred in several studies—is mechanistically unjustified by the R = E × T × C logic and predestined to fail.

### 2.9. PSDR vs. Existing Precision Oncology Frameworks

The R = E × T × C eligibility model complements existing precision oncology resources that have made major contributions to the field. OncoKB and CIViC provide curated somatic variant–drug interaction databases; PharmGKB and CPIC offer pharmacogenomic dosing guidelines for germline variants; DepMap and GDSC provide cancer dependency and drug sensitivity data across cell lines; and NCI-MATCH, TAPUR, and basket/umbrella trial designs implement biomarker-stratified enrolment in practice. PSDR’s specific contribution is an integrative eligibility framework that combines germline pharmacokinetics (E), somatic tumor genomics (T), and microenvironmental profiling (C) for repurposed agents—a combination not systematically addressed by any single existing resource. PSDR does not replace these frameworks but provides a structured approach to translating their outputs into multi-domain eligibility criteria for repurposing trial design. A comparative overview of PSDR relative to existing frameworks is provided in Table 3.

Although elements of germline pharmacogenomics, tumor genomics, and microenvironmental characterization are increasingly incorporated into contemporary precision oncology practice, these domains are typically evaluated within separate clinical, translational, or trial-design frameworks. PSDR does not claim that these concepts are absent from current oncology practice; rather, it proposes a formalized structure that integrates these domains into a single eligibility framework specifically intended for drug-repurposing studies.

The fundamental distinction is this: existing frameworks were designed for drugs developed with biomarkers in mind. PSDR—and the R = E × T × C principle it operationalizes—is designed for drugs that were not. This is a different problem, and the R = E × T × C formulation is its logical solution.

### 2.10. Limitations and Future Directions of the PSDR Framework

While PSDR provides a structured framework for integrating pharmacokinetic exposure, tumor genomics, and microenvironmental context, several limitations should be acknowledged. First, PSDR remains a conceptual and hypothesis-generating framework and has not yet undergone prospective clinical validation. The proposed multiplicative formulation (R = E × T × C) is intended to represent conditional biological logic rather than a validated quantitative prediction model. Future studies should directly compare multiplicative, additive, logistic, and machine-learning approaches to determine which framework best predicts clinical outcomes.

Second, the proposed eligibility domains are simplified representations of complex biological processes. Drug exposure, target engagement, and pathway competence exist on biological continua rather than binary states, and the thresholds used to define E, T, and C positivity will require prospective calibration for each drug–tumor combination. The current scoring system should therefore be viewed as a pragmatic operational framework rather than a definitive clinical decision tool.

Third, biomarker-enrichment strategies introduce the possibility of over-stratification. Excessively restrictive eligibility criteria may increase recruitment challenges, prolong trial timelines, increase screening costs, reduce generalizability, and potentially exclude patients who could derive benefit despite incomplete biomarker characterization. Conversely, imperfect biomarker selection may result in false-negative findings if biologically responsive subgroups are inadvertently excluded.

Fourth, implementation of PSDR depends on the availability of validated pharmacogenomic assays, tumor genomic profiling, and tumor microenvironment characterization platforms. Although many of these technologies are increasingly accessible, standardized clinical thresholds and regulatory frameworks remain under development.

Finally, biological interactions among exposure, target engagement, and microenvironmental context are likely more complex than represented by the current model. Crosstalk between these domains may introduce nonlinear effects that are not fully captured by the present framework. Future prospective studies, translational investigations, and adaptive biomarker-enriched clinical trials will be required to validate, refine, and operationalize PSDR in clinical oncology.

## 3. Drug Repurposing in Oncology: Landscape and Structural Limitations

Drug repurposing for cancer treatment has the potential to be of major benefit. From an economic perspective approved drugs have been shown safe and have achieved full human PK profiles, as well as extensive patient exposure, which can be exploited when analyzing safety data. The results obtained from such analysis can lead to safety-related indications that can be validated in a much shorter timeframe than the time consuming and costly process of de novo drug development. It has been reported that the cost for developing a new anticancer drug exceeds $2 billion per approved drug [1,11]. Hence, drug repurposing appears to be a cost-efficient strategy for cancer drug development.

Finally, from a biological perspective, many commonly used non-oncology medications affect pathways known to be important in cancer. Metformin, for example, works by activating AMPK and, via this mechanism, suppresses mTORC1 and aerobic glycolysis [29]. Statins inhibit the mevalonate pathway, reducing prenylation of Ras-family GTPases [30]. Beta-blockers attenuate sympathetic nervous system activation of the TME [14]. NSAIDs inhibit COX-2-dependent prostaglandin synthesis [31]. These mechanisms are not speculative—they are supported by preclinical data and epidemiological associations.

The structural problem is that none of these mechanistic insights have been translated into R = E × T × C eligibility criteria. Repurposing trials have consistently enrolled patients based on tumor type and performance status, applying the same eligibility logic used for cytotoxic agents. This approach is appropriate for drugs with mechanism-independent activity across heterogeneous populations. For drugs whose mechanisms are governed by the multiplicative R = E × T × C relationship, it is a systematic error.

Several additional structural limitations interact with—but are distinct from—pharmacogenomic stratification:

**Dosing inadequacy:** Most repurposed drugs were dose-optimized for non-oncological indications. Metformin doses used in diabetes trials may be insufficient to achieve intratumoral concentrations required for AMPK activation [29]. This affects the E variable directly: even in OCT1-WT patients, inadequate dosing can reduce E below the therapeutic threshold.

**Trial design limitations:** Repurposing trials frequently lack pre-specified biomarker stratification, are underpowered for subgroup analyses, and use composite endpoints designed for cytotoxic agents [32]. These limitations are compounded by the absence of R = E × T × C scoring at enrollment.

**Tumor heterogeneity:** Intra- and inter-tumoral heterogeneity in somatic mutations and TME features means that single-biopsy profiling may not capture the full T and C landscape [33]. Liquid biopsy and multi-region sampling approaches address this limitation.

**Drug potency constraints:** Some repurposed agents may lack sufficient potency for oncology applications at tolerable doses. This is a genuine biological limitation that R = E × T × C scoring cannot overcome, but it can only be properly assessed after pharmacogenomically ineligible patients have been excluded.

PSDR addresses the most systematically neglected and most directly correctable of these limitations: the absence of E, T, and C scoring at trial enrollment.

## 4. Germline Pharmacogenomics and Pharmacokinetic Adequacy

The E variable in R = E × T × C is determined by germline polymorphisms in drug-metabolizing enzymes and membrane transporters. For repurposed drugs—which were not dose-optimized for oncology—PK adequacy is the first gatekeeper of response, and its variation across patients is the most immediately correctable source of trial heterogeneity.

### 4.1. Transporter-Mediated Drug Uptake

Metformin’s entry into cells is mediated almost exclusively by organic cation transporters OCT1 (SLC22A1) and OCT2 (SLC22A2). Common OCT1 variants—R61C, G401S, M420del, G465R—reduce transport activity by 30–80% relative to wild-type. In patients carrying two reduced-function alleles, intracellular metformin concentrations may fall below the threshold required for AMPK activation, even at maximum tolerated doses [34]. In the R = E × T × C model, these patients have E ≈ 0; their inclusion in a metformin efficacy trial is mechanistically unjustified.

Statins enter hepatocytes—and hepatic tumor cells—primarily via OATP1B1, encoded by SLCO1B1. The SLCO1B1\*5 variant reduces OATP1B1 transport activity by approximately 40% and is present in 15–20% of European-ancestry populations [22]. In carriers, intratumoral statin concentrations are lower than in wild-type carriers at equivalent doses, reducing E and attenuating the downstream T × C product.

### 4.2. Metabolic Enzyme Polymorphisms

CYP2D6 metabolizes beta-blockers and SSRIs. CYP2D6 activity spans a 1000-fold range across individuals [20]. For beta-blockers, CYP2D6’s poor metabolizer status increases plasma exposure three- to five-fold—potentially enhancing E but also increasing toxicity risk. CYP2D6’s ultrarapid metabolizer status may result in subtherapeutic E, undermining the pharmacological basis for anticancer activity.

CYP2C19 governs the metabolism of several NSAIDs and SSRIs. The CYP2C19\2 and \3 loss-of-function alleles are present in 15–25% of Asian populations and 2–5% of European populations, creating substantial geographic variation in E that is relevant to global repurposing trials [35].

### 4.3. ABC Efflux Transporters and Intratumoral Drug Retention

Efflux transporters can decrease the anticancer effect of a drug by reducing its concentration in cancer cells even when the drug is well absorbed into the body. Two efflux transporters, ABCB1 (P-glycoprotein) and ABCG2, are expressed in many types of tumors and can efflux a wide variety of compounds from cancer cells. High activity of efflux transporters can decrease the concentration of anticancer drugs in tumors below the levels required for their anticancer effects. In the PSDR framework, high tumoral expression or activity of ABCB1 or ABCG2 lowers the effective intratumoral concentration of a repurposed agent and is therefore a determinant of the E variable. Where validated assays of tumor efflux transporter expression are available—by immunohistochemistry, transcriptomic profiling, or functional imaging—they should be incorporated alongside germline ADME genotyping in E scoring. Patients whose tumors are predicted to maintain therapeutic intratumoral drug exposure are scored E = 1; predicted subtherapeutic exposure due to high efflux activity corresponds to E = 0 [36].

## 5. Somatic Tumor Genomics and Target Engagement

### 5.1. LKB1/STK11 and Metformin’s AMPK Dependency

Metformin has been proposed to have cancer-suppressing effects via AMPK activation. In many tissues, including some cancer types, LKB1 is the main AMPK activator; however, lung, cervical, and pancreatic tumors lack sufficient LKB1. Loss of LKB1 is predicted to substantially reduce metformin’s cancer-suppressing effects, but it is not likely to abolish all effects completely. In addition to potential effects through pathways that involve AMPK, metformin can inhibit cancer growth and proliferation by several other mechanisms, including direct inhibition of mitochondrial complex I, lowering of insulin/IGF-1 levels, and suppression of mTORC1 through down-regulation of its main activator. The relative contribution of these additional mechanisms to cancer inhibition in the absence of LKB1 remains to be determined. The finding that the presence of LKB1 is a simple yes or no factor in predicting the efficacy of metformin is an oversimplification, as LKB1 status is a factor that modifies efficacy; therefore, patients should be grouped by their LKB1 status and the efficacy of metformin within each group determined.

### 5.2. TP53 and Statin-Induced Apoptosis

Statins inhibit HMG-CoA reductase, the rate-limiting enzyme of the mevalonate pathway, depleting downstream isoprenoid intermediates (farnesyl- and geranylgeranyl-pyrophosphate) required for post-translational prenylation of Ras- and Rho-family GTPases. Loss of prenylation attenuates membrane localization and oncogenic signaling of these GTPases and, in susceptible tumors, triggers intrinsic apoptosis [30]. Apoptotic execution in this context is largely p53-dependent: wild-type TP53 transactivates the pro-apoptotic effectors BAX, PUMA and NOXA, engaging mitochondrial outer-membrane permeabilization and caspase-9 activation [13]. TP53 is mutated in approximately 50% of human cancers, with the spectrum dominated by missense mutations in the DNA-binding domain. In TP53-mutant tumors, statin-induced apoptosis is substantially impaired (T ≈ 0 in the R = E × T × C model). The relationship is allele-specific rather than uniformly null: certain gain-of-function TP53 mutants can upregulate mevalonate pathway flux through SREBP-2 activation, potentially increasing statin sensitivity through a distinct, p53-independent mechanism [37]. Binary exclusion based on TP53 mutation status is therefore not warranted; prospective stratification by TP53 allele class (wild-type, loss-of-function, gain-of-function) is required to test the statin–TP53 dependency rigorously.

### 5.3. PIK3CA and COX-2 Pathway Selectivity

PIK3CA activating mutations create a dependency on prostaglandin E2-mediated PI3K pathway activation. COX-2-derived PGE2 activates EP receptors that signal through PI3K/AKT, creating a survival dependency that is selectively present in PIK3CA-mutant tumors [15]. NSAIDs and aspirin [38] disrupt this dependency—but only when T = 1 (PIK3CA-mutant). The PIK3CA-aspirin interaction has been validated retrospectively: HR 0.54 in PIK3CA-mutant vs. HR 1.06 in PIK3CA-WT colorectal cancer [28]. This is the most compelling empirical confirmation of the R = E × T × C principle in drug repurposing data.

## 6. Tumor Microenvironment Heterogeneity and Pathway Competence

### 6.1. Sympathetic Innervation and Beta-Blocker Response

The C variable in R = E × T × C captures TME features that determine whether the drug’s downstream mechanism has a functional substrate. For beta-blockers [39], C is determined by sympathetic innervation density. Tumors lacking TH+ sympathetic nerve fibers have no adrenergic signaling to block; C = 0 regardless of E and T [14]. In sympathetically innervated tumors, β-adrenergic signaling upregulates VEGF, recruits MDSCs, and activates pro-survival signaling—effects that beta-blockers reverse only when C = 1 [40,41].

### 6.2. Metabolic Microenvironment and Metformin Sensitivity

Metformin’s inhibition of mitochondrial complex I is selectively toxic to OXPHOS-dependent tumors. Glycolytic tumors with HIF-1α-driven Warburg metabolism can compensate for OXPHOS inhibition by upregulating glycolytic flux, setting C ≈ 0 for metformin’s primary mechanism [42,43]. Single-cell RNA sequencing can resolve tumor metabolic heterogeneity at the cell-type level, enabling C scoring for metformin eligibility assessment [44].

### 6.3. Inflammatory Microenvironment and NSAID Efficacy

COX-2 expression in the TME—particularly in tumor-associated macrophages and cancer-associated fibroblasts—determines the prostaglandin signaling landscape that NSAIDs target. In COX-2-high, prostaglandin-rich TMEs (C = 1), NSAID-mediated COX-2 inhibition reduces PGE2, shifts macrophage polarization from M2 to M1, and reduces immunosuppressive signaling [31]. In COX-2-low TMEs (C = 0), the anti-inflammatory mechanism has no substrate.

## 7. Five Critical Unmet Needs in Pharmacogenomic Drug Repurposing

The R = E × T × C equation precisely identifies five unmet needs. Each need is described in detail and relates to a missing piece of clinical practice infrastructure for measuring one of the three variables.

**Unmet Need 1: Validated Predictive Biomarker Panels for E Scoring.** Although germline pharmacogenomic panels have been developed for predictive testing for safety or dosing considerations [45], none of the existing panels have been validated for use in E scoring for oncology efficacy trials. No companion diagnostic has been FDA approved for any drug class that has been repurposed. Thus, prospective biomarker validation studies must be conducted within the framework of a phase II repurposing trial to establish the prospective criteria that define clinical utility for a test to be classified as E = 1 [46].

**Unmet Need 2: Standardized Assays for Comprehensive TME Profiling to Support C Scoring.** There is currently no uniform approach for TME characterization among or within institutions and clinical trials [47]. Currently, there are no validated C scoring assays to rigorously measure two critical features of the C infrastructure: sympathetic innervation density and tumor metabolic phenotype, which require standardized assay development and analytical validation prior to using C as an inclusion criterion [44,48].

**Unmet Need 3: Integration of EHR, T, and C Data Sources into Pharmacogenomic Registries.** Unfortunately, there is no large-scale registry that houses germline PK genotype (E), somatic tumor profile (T), TME (C), and repurposed drug response data. This type of resource would be crucial to validate the PSDR Score retrospectively and to generate hypotheses prospectively.

**Unmet Need 4: Development of Biomarker-Stratified Trial Infrastructure for PSDR Score = 3 Enrolment.** There currently exist adaptive trial platforms for single targeted agents, often for concurrent development with companion diagnostics for patient selection. For PSDR-stratified repurposing studies a specific adaptive trial infrastructure is needed.

**Unmet Need 5: Regulatory Guidance for R = E × T × C Stratification in Repurposing Trials.** There is no regulatory guidance on multi-variable pharmacogenomic stratification in repurposing trials. Regulatory guidance on the evidentiary standards for the co-development of companion diagnostic with off-patent drugs is needed.

## 8. Case Studies: Pharmacogenomic Mapping of Repurposed Drug Classes

The R = E × T × C principle provides a unifying analytical lens for interpreting the clinical record of repurposed drugs. In each case, the null results of unselected trials may reflect, in part, pharmacogenomic misclassification rather than biological implausibility but by the systematic enrollment of patients with E = 0, T = 0, or C = 0.

### 8.1. Metformin: R = (OCT1/2 Genotype) × (LKB1 Status) × (Metabolic Phenotype)

Metformin is the most extensively studied repurposed drug in oncology, with epidemiological data from diabetic cancer patients suggesting 20–40% reductions in cancer-specific mortality in multiple tumor types [49]. The MA.32 trial—the largest randomized trial of metformin in cancerenrolled 3649 unselected breast cancer patients and found no improvement in invasive disease-free survival [8,50].

The R = E × T × C analysis identifies three distinct (though potentially correlated) sources of E, T, and C = 0 in this population:

- E = 0: OCT1 LOF variants (~15% of patients) → subtherapeutic intracellular metformin;
- T impaired: LKB1/STK11 mutation (~20% of breast cancers) → AMPK-dependent mechanism substantially impaired; AMPK-independent mechanisms may partially compensate;
- C = 0: Glycolytic tumor metabolic phenotype (substantial fraction) → OXPHOS inhibition compensated.

The triple-eligible (PSDR Score = 3) subpopulation may constitute 30–50% of an unselected breast cancer cohort. Diluting this subpopulation with Score = 0 and Score = 1 patients in a 1:1 randomized trial reduces detectable effect sizes proportionally. The MA.32 trial enrolled sufficient patients to detect the signal, but enrolled them without the eligibility filter required to make the signal visible. Although inadequate pharmacogenomic stratification represents one potential explanation for the MA.32 findings, alternative explanations should also be considered. These include variability in metformin exposure, uncertainty regarding optimal anticancer dosing, biological heterogeneity across breast cancer subtypes, differences in metabolic dependency among tumors, and variability in treatment adherence. The PSDR framework is therefore proposed as one potential contributor to trial interpretation rather than a complete explanation for negative outcomes.

### 8.2. Statins: R = (SLCO1B1 Genotype) × (TP53 Status) × (Mevalonate Dependency)

Statins generate compelling observational data: retrospective analyses associate statin use with 20–40% reductions in cancer-specific mortality in colorectal, breast, and prostate cancer [7,51]. Interventional trials have consistently failed. The R = E × T × C analysis explains why: in an unselected cancer population, TP53 is mutated in ~50% of tumors (T = 0) and SLCO1B1\*5 reduces E in ~15–20% of patients. The PSDR Score = 3 fraction is approximately 40–42%. Statin trials in unselected populations test a drug in a majority Score ≤ 1 population by design.

### 8.3. Beta-Blockers: R = (ADRB2 Genotype) × (β2-AR Expression) × (Sympathetic TME Density)

Beta-blockers present a unique R = E × T × C profile because the C variable—sympathetic innervation density—is the dominant determinant of response eligibility. Retrospective data show heterogeneous beta-blocker effects across tumor types and studies [6,39,41], consistent with C-variable heterogeneity across patient populations. Prospective trials stratifying by TH+ sympathetic nerve density and ADRB2 genotype would test the R = E × T × C hypothesis with appropriate rigor.

### 8.4. NSAIDs and Aspirin: R = (CYP2C9 Genotype) × (PIK3CA Mutation) × (COX-2/TME)

The PIK3CA-aspirin interaction represents the most advanced empirical validation of the R = E × T × C principle in drug repurposing. The HR 0.54 in PIK3CA-mutant vs. HR 1.06 in PIK3CA-WT colorectal cancer [28] is precisely what the multiplicative model predicts: when T = 0 (PIK3CA-WT), R = 0 regardless of E and C; when T = 1 (PIK3CA-mutant), the E × C product determines response magnitude. The Add-Aspirin trial (NCT02804815) provides an existing platform for prospective validation.

### 8.5. SSRIs: R = (CYP2D6 Genotype) × (Putative CRBN-IKZF Pathway) × (B-Cell Lymphoma TME)—Speculative Case Study

SSRIs have been reported to exhibit direct signaling effects on lymphoma cells [52], and a CRBN-mediated IKZF degradation mechanism has been proposed by analogy with immunomodulatory drugs (IMiDs/CELMoDs). However, CRBN-mediated IKZF1/3 degradation is an established mechanism for lenalidomide, pomalidomide, and next-generation CELMoDs not for SSRIs, for which direct CRBN-binding evidence is limited. The SSRI case study is therefore presented as a hypothesis-generating example requiring primary mechanistic validation. The R = E × T × C analysis restricts this speculative mechanism to CYP2D6-adequate, CRBN-intact, IKZF-dependent B-cell malignancies—but this restriction should not be interpreted as clinical guidance until the mechanism is experimentally confirmed.

## 9. Enabling Technologies

Three technological platforms are transforming the feasibility of R = E × T × C scoring in clinical trials.

### 9.1. CRISPR Functional Genomics

Genome-scale CRISPR loss-of-function screens can identify T and C variable determinants for repurposed drug candidates at scale. By systematically knocking out individual genes across cancer cell lines treated with repurposed agents, CRISPR screens identify somatic dependencies and TME interactions that determine drug sensitivity—generating T and C scoring hypotheses that are directly translatable to trial design [24,26]. The Cancer Dependency Map (DepMap) provides CRISPR screen data across 1000+ cancer cell lines; integrating these data with germline PK modeling generates complete R = E × T × C predictions for novel repurposing candidates.

### 9.2. Single-Cell Transcriptomics

Single-cell RNA sequencing resolves the cellular heterogeneity of the TME at a resolution that bulk sequencing cannot achieve—enabling precise C scoring. For PSDR, single-cell approaches identify: (1) tumor cell subpopulations with specific metabolic dependencies (C for metformin); (2) immune cell infiltration patterns relevant to immunomodulatory drug mechanisms (C for immunotherapies); (3) stromal cell types expressing drug targets (C for NSAIDs); and (4) sympathetic nerve fiber-associated gene signatures (C for beta-blockers) [44].

### 9.3. AI-Driven Biomarker Discovery

AI-driven multi-variable analysis can identify R = E × T × C predictors of drug response that are not apparent from single-variable analyses. The models can incorporate germline genotype (E), somatic mutation (T), gene expression (C) and treatment outcome to identify the best predictors of response. In addition, structured prior knowledge (drug–target interaction networks: STITCH, DrugBank; pharmacogenomic databases: PharmGKB, CPIC) can be used to inform and constrain the models.

## 10. Clinical Translation Strategies

### 10.1. Biomarker-Stratified Trial Designs for PSDR Score = 3 Enrolment

For R = E × T × C repurposing trials, three different study designs can be distinguished.

**Enrichment design:** Only patients classified as PSDR Score = 3 are enrolled, maximizing statistical power by restricting the study population to those most likely to respond.

**Stratified design:** All patients are enrolled but PSDR Score subgroup analyses are pre-specified as co-primary endpoints, allowing evaluation of both overall and biomarker-selected populations.

**Biomarker-adaptive design:** Interim R = E × T × C data are used to modify enrolment criteria, allowing the trial to focus progressively on the PSDR Score = 3 subpopulation as evidence accumulates.

### 10.2. Pharmacogenomic Registries for R = E × T × C Validation

The pharmacogenomic registry would track data for repurposed drugs in oncology by tracking E (germline PK genotype), T (somatic tumor profile), C (TME), and drug exposure and outcomes. This data would be used to generate retrospective hypotheses to test and validate for improved PSDR scoring scores for oncolytic efficacy. There are many examples of analogous registries in pharmacogenomics such as CPIC (https://cpicpgx.org/ accessed on 12 May 2026) for predicting safe and adverse effects of drugs and PharmGKB (https://www.pharmgkb.org/ accessed on 29 May 2026) for interactions between drugs and genes. There are none, however, focused on oncology drug repurposing.

## 11. Future Directions

The R = E × T × C principle and the PSDR framework it governs define a research agenda with several high-priority directions:

Prospective PSDR Score validation: Each of the five testable hypotheses in Section 2.8 requires prospective validation in PSDR Score = 3 enriched cohorts. The PIK3CA-aspirin hypothesis is most advanced and could be validated within the Add-Aspirin trial. The OCT1-LKB1-metformin hypothesis is supported by sufficient mechanistic and retrospective evidence to justify a dedicated phase II PSDR-stratified trial.

Continuous R = E × T × C modeling: The discrete 0/1 scoring system is a clinical simplification. Future work should develop continuous E, T, and C scoring functions—incorporating quantitative PK predictions from PBPK modeling, tumor genomic pathway activity scores, and quantitative TME profiling—to generate continuous R predictions that better capture response probability distributions.

Computational PSDR scoring for novel candidates: Machine learning models trained on existing pharmacogenomic databases [53] could generate R = E × T × C scores for novel repurposing candidates, prioritizing drugs with the strongest predicted Score = 3 subpopulations for clinical testing [54]. This extends PSDR from a framework for evaluating known candidates to a tool for prioritizing new ones [55].

Regulatory engagement: Regulatory agencies have not issued guidance specific to multi-variable pharmacogenomic stratification in repurposing trials. The existing FDA enrichment strategy guidance provides a starting framework [56]; R = E × T × C-specific guidance would accelerate implementation and establish evidentiary standards for companion diagnostic co-development with off-patent agents [57].

Extension to combination repurposing: The R = E × T × C principle extends naturally to drug combinations. For a two-drug repurposing combination, the joint response probability is R_12_ = (E_1_ × T_1_ × C_1_) × (E_2_ × T_2_ × C_2_) × I, where I captures drug–drug interaction effects on the shared pathway. This formulation generates specific predictions about which combinations are synergistic in which patient subpopulations—a level of precision that current combination repurposing trials do not approach.

## 12. Conclusions

Drug repurposing in oncology has had limited clinical success to date, with failures attributable to multiple factors including dosing, potency, trial design, patient heterogeneity, and absence of pharmacogenomic stratification. The drugs had an on-target mechanism of action within the intended cancer pathway, and indeed they can and do treat some patients with the disease. Unselected trial designs may reduce the likelihood of detecting benefit in molecularly responsive patient subsets, particularly when treatment response depends on pharmacogenomic factors. The failure is that those patients have never been identified in clinical trials, primarily because the logic as to patient eligibility for study of these compounds has never been seriously applied.

The relationship between a drug and its effect on an individual can be described by the equation R = E × T × C, where R is the effect of the drug, E is the exposure to the drug, T is the engagement of the drug with its intended target, and C is the context of the drug’s effect on a particular biological pathway. Each domain is conceptually separable but biologically interacting with the others, and must be non-zero for any effect of the other variables to be greater than zero. This is not a novel hypothesis about pharmacogenomics, but rather a logical consequence of how drugs affect biology at the molecular level, a property that has been well understood by pharmacologists for decades. PSDR applies this property to a major class of cancer drugs for which such an analysis has not been performed previously.

The implications of these findings are extremely practical. It is not possible to do a clinical trial on an unscreened patient population (no R = E × T × C stratification) and obtain any meaningful efficacy information (positive or negative). This is because the patients enrolled in such a trial will represent an undefined mixture of molecular subpopulations, and the results of the trial cannot be used as evidence of the lack of efficacy of a given therapy. In contrast, identification of drug activity in any of the Score = 3 subpopulations defined by the PSDR should constitute strong scientific evidence that should be explored prospectively.

From a clinical infrastructures and assay technologies viewpoint, as well as from trial designs and regulatory issues, there are no barriers to performing PSDR-stratified repurposing trials. Coordinated deployment of these existing resources is both feasible and necessary if repurposing trials are to yield interpretable efficacy data. The R = E × T × C formulation is offered as a pharmacogenomic eligibility framework specific to oncology drug repurposing, not as a generalized predictive equation beyond this context.

There is preclinical evidence and also epidemiological evidence in human populations that drug repurposing can be very effective for many types of cancer. However, there is no translation to clinical success. What is missing is systematic pharmacogenomic stratification of patients to progress from signal to proof of concept (E = 1, T = 1, C = 1).

Excessive biomarker stratification may create practical challenges, including prolonged recruitment periods, increased screening costs, reduced generalizability, and the risk of excluding potentially responsive patients because of imperfect biomarker classification. Future studies will need to balance biological enrichment against feasibility and external validity.

## 13. Limitations

The PSDR framework has several important limitations that must be acknowledged before clinical or regulatory application. Conceptual, not empirical: R = E × T × C is a conceptual eligibility model, not an empirically validated quantitative law. The framework has not been prospectively tested in a clinical trial, and its predictive performance has not been compared against additive models, logistic regression, Cox proportional hazards models, Bayesian biomarker–response models, or established PK/PD frameworks (e.g., Bliss independence, Loewe additivity). Such comparisons are required before the multiplicative structure can be claimed to outperform simpler models. Biological correlation of domains: E, T, and C are treated as conceptually distinct domains, but they are biologically correlated. Tumor genotype (T) shapes microenvironmental features (C); inflammatory signaling (C) can regulate transporter expression (E); metabolic phenotype (C) interacts with target pathway activity (T). Treating the domains as independent may underestimate their interdependence and introduce estimation bias in multi-domain scoring. Biomarkers as imperfect proxies: The biomarkers used to score E, T, and C are proxies for underlying biological processes. OCT1/2 genotype predicts but does not guarantee intracellular drug concentration; LKB1/STK11 mutation status predicts but does not fully determine AMPK pathway activity; TME features assessed on a single biopsy may not represent tumor-wide heterogeneity. Biomarker imprecision introduces noise into eligibility scoring. Binary scoring is a simplified approximation: The 0/1 binary scoring system is a practical simplification of what are, in reality, continuous biological variables. Threshold values for E, T, and C eligibility are currently uncertain and have not been prospectively calibrated. Score 2 (two of three domains met) is assigned to an exploratory cohort only; it should not be used as a primary efficacy cohort without further validation. Drug-specific hypotheses require individual validation: Each of the five drug hypotheses (metformin, statins, beta-blockers, NSAIDs, SSRIs) requires independent prospective validation. The SSRI hypothesis in particular relies on a CRBN–IKZF mechanism that is established for IMiDs/CELMoDs but not directly demonstrated for SSRIs; it is presented as hypothesis-generating only. No empirical model comparison: The PSDR framework has not been compared against existing precision oncology resources (OncoKB, CIViC, PharmGKB, CPIC, DepMap, GDSC) in a head-to-head predictive performance analysis. The claim that PSDR adds value beyond these resources requires empirical demonstration. Sample size and power: Formal power calculations for PSDR-stratified trials have not been performed. Required sample sizes depend on biomarker prevalence, expected effect size, endpoint selection, and acceptable type I/II error rates, all of which are drug- and indication-specific.

## Figures and Tables

**Table 1:** Interpretation of the PSDR eligibility scoring system.

Score	Interpretation
3 (E = 1, T = 1, C = 1)	All conditions met → Enroll in primary efficacy cohort
2 (any one scored 0 or uncertain)	One condition absent → Exploratory cohort only
1 (any two = 0)	Two conditions absent → Exclude from efficacy analysis
0 (all = 0)	No conditions met → Exclude

**Table 2:** PSDR eligibility criteria for five repurposed drug classes. Each drug class is mapped to its E (Exposure/germline ADME), T (Target/somatic genomics), and C (Context/TME) variables with the PSDR Score = 3 eligibility criteria.

Drug Class	E Variable (Germline ADME)	T Variable (Somatic Genomics)	C Variable (TME Context)	PSDR Score = 3 Criteria
Metformin	OCT1/2 WT (SLC22A1/A2)	LKB1/STK11 WT	OXPHOS-dependent metabolic phenotype	OCT1 WT × LKB1 WT × OXPHOS+
Statins	SLCO1B1 WT (*1/*1)	TP53 WT	Mevalonate-dependent tumor	SLCO1B1 WT × TP53 WT × Mev+
Beta-blockers	ADRB2 Arg16 genotype	β2-AR expressed on tumor/immune	High TH+ sympathetic nerve density	ADRB2 Arg16 × β2-AR+ × TH+
NSAIDs/Aspirin	CYP2C9 WT	PIK3CA activating mutation	COX-2 overexpression in tumor/stroma	CYP2C9 WT × PIK3CA-mut × COX-2+
SSRIs	CYP2D6 IM/EM	CRBN WT + IKZF1/3-dependent	B-cell lymphoma microenvironment	CYP2D6 IM/EM × CRBN WT × B-cell

1 SLCO1B1 WT (*1/*1) denotes the wild-type genotype associated with normal transporter function and standard statin pharmacokinetics.

**Table 3:** Comparison of PSDR with existing precision oncology frameworks in terms of pharmacokinetic exposure (E), tumor genomics (T), and microenvironmental context (C).

Framework	Primary Variable	Germline PK (E)	Somatic Genomics (T)	TME Context (C)	Applicable to Repurposed Drugs
PSDR (R = E × T × C)	All three (multiplicative)	Required	Required	Required	Designed for repurposed drugs
Precision oncology (e.g., EGFR)	Somatic mutation (T)	Not assessed	Primary variable	Not assessed	Designed for de novo targeted agents
Pharmacogenomics (PGx)	Germline variant (E)	Primary variable	Not assessed	Not assessed	Partial (E only)
Immunotherapy biomarkers	TME (PD-L1, TMB, MSI)	Not assessed	Partial	Primary variable	Partial (C only)
Adaptive enrichment designs	Biomarker TBD	Variable	Variable	Variable	Design-agnostic

## Data Availability

No new data were created or analyzed in this study.
